# Genetic and Epigenetic Events Generate Multiple Pathways in Colorectal Cancer Progression

**DOI:** 10.1155/2012/509348

**Published:** 2012-07-24

**Authors:** Massimo Pancione, Andrea Remo, Vittorio Colantuoni

**Affiliations:** ^1^Department of Biological, Geological and Environmental Sciences, University of Sannio, Via Port'Arsa 11, 82100 Benevento, Italy; ^2^Department of Pathology, “Mater Salutis” Hospital, ULSS21 Legnago, Verona, Italy

## Abstract

Colorectal cancer (CRC) is one of the most common causes of death, despite decades of research. Initially considered as a disease due to genetic mutations, it is now viewed as a complex malignancy because of the involvement of epigenetic abnormalities. A functional equivalence between genetic and epigenetic mechanisms has been suggested in CRC initiation and progression. A hallmark of CRC is its pathogenetic heterogeneity attained through at least three distinct pathways: a traditional (adenoma-carcinoma sequence), an alternative, and more recently the so-called serrated pathway. While the alternative pathway is more heterogeneous and less characterized, the traditional and serrated pathways appear to be more homogeneous and clearly distinct. One unsolved question in colon cancer biology concerns the cells of origin and from which crypt compartment the different pathways originate. Based on molecular and pathological evidences, we propose that the traditional and serrated pathways originate from different crypt compartments explaining their genetic/epigenetic and clinicopathological differences. In this paper, we will discuss the current knowledge of CRC pathogenesis and, specifically, summarize the role of genetic/epigenetic changes in the origin and progression of the multiple CRC pathways. Elucidation of the link between the molecular and clinico-pathological aspects of CRC would improve our understanding of its etiology and impact both prevention and treatment.

## 1. Background: The Molecular Basis of Colon Carcinogenesis

Colorectal cancers (CRCs) arise through a multistep process in which genetic and epigenetic alterations accumulate in a sequential order. Three different pathogenetic pathways have been implicated in the development of these tumors: (1) chromosomal instability (CIN); (2) microsatellite instability (MSI); (3) CpG island methylator phenotype (CIMP).

The CIN pathway is associated with the sequential deregulation of tumor suppressor genes (TSGs) and oncogenes such as, *APC*, *KRAS*, *DCC/SMAD4*, and *TP53*. It generally occurs within inherited tumors, such as familial adenomatous polyposis (FAP), but it has also been associated with the majority of sporadic CRCs. Microsatellite instability is responsible for the Lynch syndrome and sporadic tumors and is mainly caused by inactivation of the DNA mismatch repair genes (*hMLH1*, *hMSH2*, *hMSH6*, and *hPMS2*). CRCs displaying MSI tend to be rightsided, generally have high histological grades, a mucinous phenotype, and diagnosed at lower pathological stages than CIN cancers. The CpG island methylator phenotype (CIMP) refers to the widespread hypermethylation of CpG islands at several loci. Typically, one type of molecular signature predominates suggesting that the three pathways rarely overlap; a complex interplay occurs in some tumors whereby one pathway is a result of one other (i.e., CIMP and MSI due to *hMLH1* promoter hypermethylation).

## 2. Introduction

More than one million of individuals develop CRC every year worldwide [1, 2]. Despite the remarkable accomplishments in new therapeutic options, CRC remains one of the most common malignancies [3, 4]. Intriguingly, during the last decade, CRC incidence in the Asian population increased from two- to four-times, whereas it progressively diminished in western countries, implying yet undefined gene-environment interactions [4, 5]. CRC occurs sporadically in the majority of cases and only in 5–10% is due to inherited mutations in well-known cancer-related genes [6]; about 25% of patients, however, reveal a family history of the disease, suggesting a specific contribution by yet unidentified genes [6]. In 1990, Fearon and Vogelstein proposed a model whereby CRC proceeds through a series of morphological steps due to specific genetic alterations [7]. The model emphasizes the central role of the adenomatous polyp as the precursor lesion and provides evidence that in the majority of CRCs the primary event is the aberrant activation of the APC/*β*-catenin pathway, followed by *RAS/RAF* mutations and loss of p53 function at later stages [7]. Ten years later, only 7% of CRCs have been shown to bear mutations in all three genes, implying that multiple pathways may be involved in the tumorigenic process [8]. Recently, the development of CRC has been considered from a different point of view [9–12]. Genetic alterations are, in fact, only a piece of a more complex puzzle [13]; epigenetic variations in cancer-related genes and noncoding RNAs play also a role contributing to the malignant status [14, 15]. The timing and combinations of genetic and epigenetic events rather than the mere accumulation of genetic disorders appear to confer cancer cells a selective advantage resulting in the activation of distinct pathways [11, 14, 16]. Genomic instability is emerging as a fundamental process in colorectal tumorigenesis [17], as highlighted by a number of inherited CRCs such as FAP, MYH associated polyposis (MAP), and hereditary nonpolyposis colon cancer (HNPCC). They are caused, in fact, by germline mutations in cancer-related genes involved in DNA duplication or repair, respectively [2, 6]. CIN, a hallmark of these events, is a process that generates gene deletions, duplications, and chromosomal rearrangements [18]. CRC development has also been associated with frequent mutations at simple sequence repeats or microsatellites, generating MSI [19]. Recently, it has been hypothesized that point mutation instability (PIN), a process that increases spontaneous mutations in random nucleotide sequences, could contribute to both CIN and MSI [20, 21]. In the last decade, a novel type of instability has been suggested to influence CRC pathogenesis. It is merely epigenetic and referred as CIMP [22–24]. Current evidences indicate that only one type of genomic instability predominates, providing the main “genetic or epigenetic signature” to the development of a specific CRC [23]. Although the molecular bases of genomic instability remain elusive, the global genomic/epigenomic aberrations differentially mark three distinct developmental pathways: traditional, alternative, and serrated [10, 12]. This suggests that not a single but multiple pathogenetic mechanisms account for colorectal tumorigenesis. One attractive question is to establish the relative contribution of each of these pathways to tumor development and the effects they exert on the phenotype and clinical behaviour. In this paper we will examine the achievements in our understanding of CRC pathogenesis with a special focus on the molecular basis of its heterogeneity.

## 3. Genomic and Epigenomic Instability ****Associate with Different CRC ****Pathogenetic Pathways

**Figure 1:**
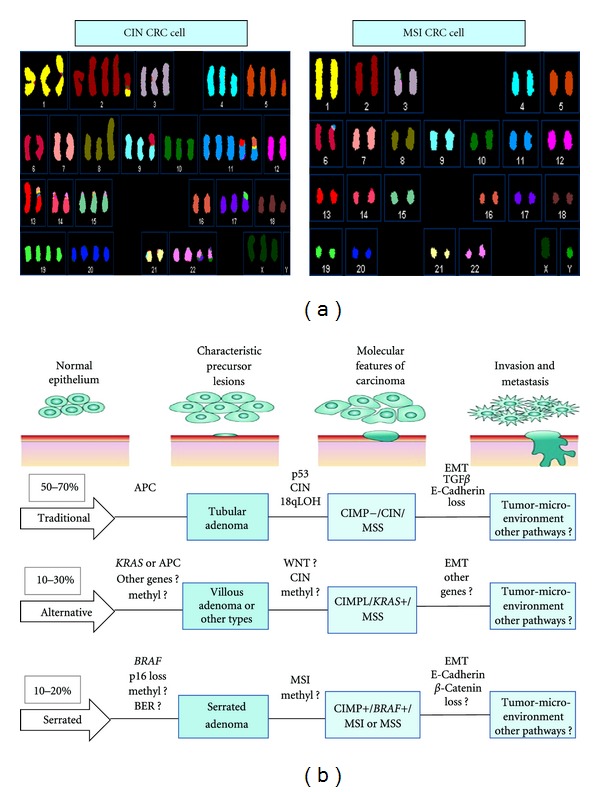
Genomic instability and multiple pathways in colorectal cancer pathogenesis. (a) Comparison of the numerical chromosomal abnormalities between two representative colon cancer cell lines characterized by CIN (highly rearranged aneuploid karyotype) and MSI (diploid karyotype), respectively. The images are publicly available on the web at: http://www.path.cam.ac.uk. (b) Three distinct parallel pathways with the approximate indicated prevalence are implicated in colon cancer pathogenesis: traditional, alternative, and serrated. The sequential of genetic and epigenetic changes occurring in each pathway are simplified, along with the characteristic precursor lesions (adenomas) and distinctive molecular features of the corresponding carcinomas. The traditional and serrated pathways are more homogeneous and clearly distinguishable; the alternative is more heterogeneous. The best known genetic/epigenetic alterations are indicated in bold, the poorly understood or hypothetical pathways are indicated in italics. APC, adenomatous polyposis coli; MSI, microsatellite instability; CIMP, CpG island methylator phenotype; CIMPL, CIMP-low; CIN, chromosomal instability; MSS, microsatellite stable; BER, base excision repair pathway; Methyl, DNA methylation silencing in yet unknown genes; WNT, wingless pathway; EMT, epithelial mesenchymal transition; TGF*β*, transforming growth factor-beta; LOH, loss-of-heterozigosity. Note: tumor microenvironment indicates the crosstalk between cancer cells and cells of the neoplastic stroma.

Genomic stability is strictly controlled in order to maintain cell homeostasis [17]. Hence, any defect in the mechanisms governing this event will promote mutational processes, selection, and clonal expansion of the mutated cells, contributing to cancer progression. CIN is the most common type of genomic instability occurring in 60% of CRCs, mainly in tumors proficient in DNA mismatch repair [18]. CIN positive tumors are characterized by frequent loss-of-heterozygosity (LOH) at tumor suppressor gene loci, chromosomal rearrangements, and numerical abnormalities “manifested as aneuploidy” (Figure 1(a)). MSI accounts, instead, for approximately 15–20% of sporadic CRCs with a well-defined phenotype resulting from loss of DNA mismatch repair functions [19]. The characteristic signature of MSI is the deletion of one element in a repetitive region of DNA that generates a frameshift in the coding sequence and hence gene inactivation, generally at tumor suppressor genes loci [19]. Interestingly, most MSI-CRCs are apparently diploid or near-diploid (Figure 1(a)). In association with mutations in cancer-related genes, CIN has been proposed for long time as the driving force to achieve the malignant status [7]. During the last decade, also epigenetic events, that is heritable changes in gene expression not accompanied by changes in the DNA sequence, have been recognized to play a crucial role in CRC development [25]. The term encompasses histone modification, nucleosome location, noncoding RNAs, and DNA methylation [13, 14]. DNA hypermethylation at specific regulatory sites, enriched in CpG motifs (CpG islands) in the promoter regions of tumor suppressor genes, has been linked to transcription repression in human tumors [14]. In 1999, Toyota et al. proposed the term CIMP to describe a subset of CRCs that consistently show widespread CpG island hypermethylation at seven different loci defined methylated in tumors (MINT) [22]. Subsequently, methylation at least three MINT loci has strongly been correlated with *CDKN2A *(p16) and *hMLH1* methylation constituting the so-called “classic panel” and providing a simplified approach to CIMP definition [22–25]. Using these markers, CIMP positive tumors are more frequently associated with MSI-CRCs than the MSS counterpart and localized to the right colon (up to 40%) than left colon and rectum (3–12%). The CIMP phenotype is, however, uncommon in HNPCC that exhibits MSI, suggesting distinct underlying molecular processes [24–26]. The existence of such a phenotype has largely been debated and a consensus on which markers should be used for its definition has not been reached yet. To overcome this difficulty and support the CIMP phenotype as a distinct CRC molecular trait,Curtin et al.proposed alternative markers (*CACNA1G, IGF2, NEUROG1, RUNX3 *and *SOCS1) *to the classic list of genes [26]. Based on this new panel, CIMP positive tumors do not have any relationship with *KRAS *mutations but strongly correlate with the *BRAF* V600E mutation [25, 26]. According to the number of methylated CIMP markers, CRCs can be divided in three epigenotypes: CIMP-high, -low and -negative [9]. CIMP positive tumors are also referred as CIMP-high CRCs [26]. The CIMP phenotype has recently been validated as an independent pathway using a whole-methylome sequencing approach [27–29]. CIMP tumors show differentially methylated CpG sites as compared to CIMP negative tumors and normal matched controls. Many of these CpG sites appear to be more prone to methylation and uniquely methylated in CIMP tumors with respect to non-CIMP tumors [27–29]. Moreover, DNA methylation spreading appears to be preferentially unidirectional and likely due to the binding of specific transcription factors [27]. According to Berman et al., the hypermethylated areas of CIMP tumors are focal (the so-called partially methylated domains (PMDs)) and associated with long-range hypomethylated regions [29]. Consequently, the CRC genome can be divided into four clusters: (1) methylation-prone, that is, regions “methylated in tumor,” but not in normal tissues; (2) methylation resistant, that is, regions “no methylated in either cell type;” (3) methylation-loss, that is regions “methylated in normal, but not in tumor tissue;” (4) constitutively methylated loci, that is, regions “methylated either in normal and in tumor tissue.” Interestingly, the methylation prone elements are highly enriched for marks with polycomb repressive complex 1 and 2 activity, resembling human embryonic stem cells (hES) [27, 29]. These studies support the notion that widespread DNA methylation changes in colon cancer are linked to specific silencing programs, suggesting the CIMP phenotype as part of a specific pathway of intense DNA hypermethylation, defined “epigenomic instability” [25–29].

**Figure 2:**
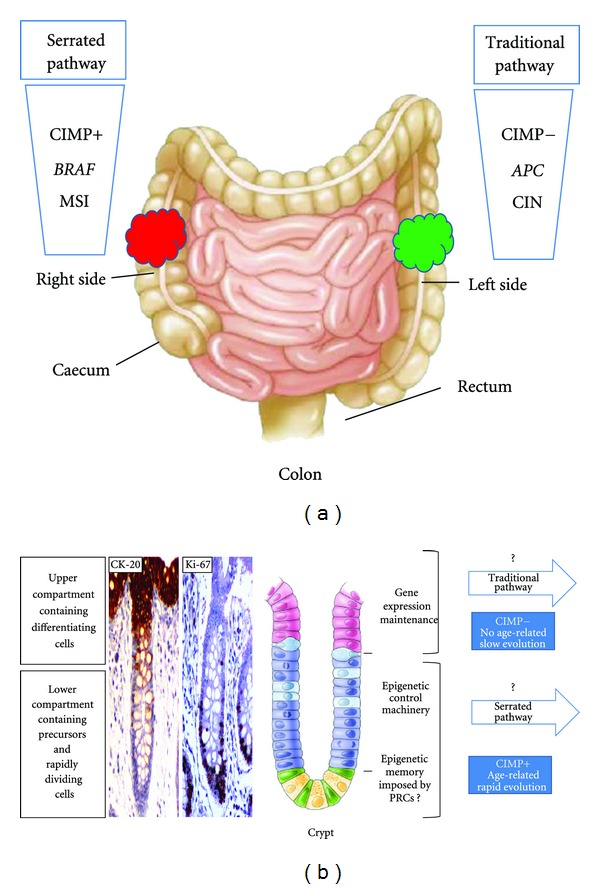
Epigenetic alterations may differentiate the traditional and serrated pathway at early stage of tumor development. (a) The serrated and traditional pathways show clinical differences, especially when referred to the site of origin of the tumor. The serrated pathway tends to be localized to the proximal (right) colon; the traditional to the distal (left) colon. The main molecular alterations that clearly distinguish the two pathways are the presence (+)/absence (−) of the CIMP phenotype and different genetic characteristics (*BRAF*/MSI versus *APC*/CIN). (b) The colonic epithelium consists of spatially separated, nonproliferative/differentiated cells at the tip of the villi, marked by cytokeratin 20 and proliferative/undifferentiated cell populations marked by Ki67. The molecular mechanisms underlying CIMP and CIN are still unknown; however, these alterations may evolve in a nonrandom fashion. According to the top-down model [31], the traditional pathway may arise from genetic lesions (*APC *mutations) confined to the upper crypt compartment. In contrast, the serrated pathway may originate in the lower crypt compartment by yet uncharacterized genetic and/or epigenetic lesions. Current evidences support the idea that the specific functions played in the lower compartment are maintained by an epigenetic program finely regulated by PRCs [13, 32–35]. Initial lesions in cells of this compartment may predispose to the epigenetic characteristics of the adult cancer through an “epigenetic memory.” This may explain why these specific precursor lesions proliferate downward or laterally, are age-related, rapidly progressive, and prone to CIMP. APC, adenomatous polyposis coli; MSI, microsatellite instability; CIMP, CpG island methylator phenotype; CIN, chromosomal instability; polycomb repressive complexes (PRCs).

Based on these new concepts, Issa revised the model of CRC development: instead of a linear progression of single events, he proposes three distinct multiple pathways, each of which based on different molecular mechanisms and variable prognosis [30]. These pathways are illustrated in (Figure 1(b)) that takes into consideration the current understanding and the novel hypotheses. Out of the three pathways proposed, the alternative one is the most heterogeneous as it originates mainly from villous but also from serrated adenomas (Figure 1(b)). It is characterized by a CIMP-low phenotype, predominant *KRAS* but occasional *BRAF* mutations, and no CIN and is associated with a worse prognosis [9, 10, 30]. In contrast, the traditional and serrated pathways appear to be clearly distinct (Figure 2(a)). Based on their molecular and pathological characteristics, the major distinction between the serrated and traditional pathways resides in their epigenomic instability (CIMP positive versus negative) and subsequently in gene alterations (*BRAF* versus *APC*, MSI versus CIN) (Figure 2(a)). We propose that the specific identity of these two pathways is established at an early evolutionary stage and fully enforced within precancerous lesions. Tumor development through the traditional pathway is, in fact, relatively slow (5–20 years), probably due to the fact that the initial events occur in the fully differentiated cells of the colonic crypt (Figure 2(b)). APC mutations, generally, are detected in the cells of the upper crypt compartment according to the top-down morphogenetic model [31]. The causal events underlying the serrated pathway, instead, may take place in the cells of the lower crypt compartment, whose functions are finely regulated by epigenetic mechanisms carried out by the components of the PcG polycomb repressive complexes (PRCs) [13, 32–34]. An “epigenetic memory” might operate at this compartment predisposing to the epigenetic characteristics of the adult cancer cells [32–35]. Albeit speculative, the present model integrates both morphological and molecular evidences and would explain why the precursor lesions of the traditional pathway originate in the upper part of the crypt tend to grow upward while those of the serrated pathway grow downward or laterally, are rapidly progressive and prone to CIMP (Figure 2(b)) [12, 30, 36]. Among the genetic alterations associated with these events,* BRAF* mutations have been proposed as the earliest genetic event, followed by inactivation of p16/INK4a [36, 37]. Whether this is the seminal genetic lesion and which are the subsequent ones occurring in serrated adenomas remain to be established (Figure 1(b)). Also the nature and characteristics of the tumor-initiating cells (TICs) from which the different pathogenetic pathways originate are not known yet, although they are the focus of intense research [34, 38]. TICs are able to form a malignant stem-cell compartment with a hierarchical organization and a specialized microenvironment “niche,” resembling the normal stem-cell system at the bottom of the crypt (Figure 2(b)). TICs are constituted by at least three different subtypes and only one of them has recently been demonstrated to be truly tumorigenic [38]. Cells of this subfraction are defined long-term TICs (LT-TICs), because they can initiate tumor formation, maintain self-renewal, and promote distant metastasis [38]. Genetic heterogeneity does not significantly contribute to the functional differences between distinct types of TICs. As mentioned above, the methylation profile of hypermethylated regions in CIMP tumors appears to be very similar to normal stem cells, as it is enriched in repressive marks of the PRC family and in specific transcription factors. The relative extent of CpG island hyper- and hypomethylation in tumors may reflect different features of the TICs subclone of origin (i.e., long-term stem cells versus transient amplifying precursors or differentiated cells). We hypothesize that the early appearance of CIMP and higher CpG island hypermethylation present in the serrated pathway could be related to the specific chromatin organization program of the cell of origin (Figure 2(b)). The “hierarchy model” hypothesizes that cancer stem cells initiate the malignant process and provide a continuous source of transformed cells expanding the tumor mass and tumor heterogeneity. Moreover, they display an increased ability to survive genotoxic stress and injury, suggesting that they are responsible for chemo- and radioresistance, for metastasis and, ultimately, patient demise [38]. The discovery of different types of TICs suggests a distinct contribution of the stem-cell-like population to tumor formation and progression; however, the molecular link between the “cell of origin” and the specific pathogenetic pathway remains to be demonstrated [13, 30, 34, 38]. Cancers related to chronic inflammation processes, such as colitis-associated cancers (CAC), follow an alternative pathway, generally initiated by mutations in *PT53* or by COX2 overexpression and followed by *APC* inactivation in later stages [39].

Although these pathways represent a simplification of our current knowledge on colon tumorigenesis, they provide evidence to support a revision of the classical adenoma-carcinoma sequence. The complete elucidation of the molecular modifications underlying these multiple pathogenetic pathways, in association with lifestyle and genetic polymorphisms, will likely change our understanding of the molecular basis of colorectal tumorigenesis.

## 4. Chromatin Remodeling and Epigenetic ****Abnormalities in Colon Carcinogenesis

Epigenetic events play a role in tumorigenesis as they activate oncogenes and inactivate tumor suppressor genes according to the multistep origin of the process [13, 14]. They do not involve changes in DNA sequences but rather are self-propagating and potentially reversible molecular signatures [13, 14]. The notion of epigenetic variations of DNA along with the identification of the CIMP phenotype and, more recently, the discovery of various classes of noncoding (ncRNA) and micro-RNAs (miRNA) have significantly modified our knowledge on carcinogenesis [27, 40, 41]. Gene silencing occurring after DNA hypermethylation is a complex process mediated either by direct inhibition of transcription factors binding or by methyl-DNA binding proteins (MBD) [42]. These latter proteins, in turn, recruit other transcriptional repressors such as histone deacetylases (HDACs) and histone methyl transferases (HMT), generating a transcriptionally inactive chromatin [42, 43]. Nucleosome positioning is also relevant in determining accessibility of transcription factors to their target DNA sequences [44]. A series of protein complexes, known as chromatin remodelers, mediate this event as they can slide, destabilize, or relocate nucleosomes in an ATP-dependent manner [44, 45]. The SWI/SNF mating-type switching (SWI) and sucrose nonfermenting (SNF) subfamily has particularly been investigated in cancer research [46]. The complex contains two subunits with ATPase activity, Brahma-(Brm), Brahma-related gene-1 (*BRG1*) and several Brahma associated factors (BAFs) (Table 1) [44–46]. Loss-of-function of SWI/SNF components impairs normal chromatin remodeling in human cancers [46, 47]. *BRG1 *is mutated in several cancer cell lines including those derived from colon [48], and loss of *BRG1* is observed in a wide variety of solid tumors [49]. Mice heterozygous for mutations at *Brg1 *are cancer-prone; the precise role of *Brg1* in CRC is, however, still controversial [48–53]. Loss of *Brm* expression is observed in several tumors and appears to occur at the posttranscriptional level [46, 49]; recent evidences show that *Brm* promotes the differentiation of gastric cancer cells [54]. SNF5 (also called SMARCB1, INI1, BAF47), localized to the 22q11.2 chromosome region, is the most extensively studied subunit of the SWI/SNF complex for its critical role in tumorigenesis (Table 1) [46, 55]. In fact, *SNF5 *is inactivated either at germline or at somatic level in malignant rhabdoid tumors (MRTs), a pediatric and highly lethal neoplasm of the kidney and brain [55–58]. Despite their extremely aggressive behaviour, most of these tumors display a normal karyotype and the so-called rhabdoid cells [58]. They are characterized by an eccentrically located and large nucleus, prominent nucleoli, eosinophilic cytoplasm with a spheroid perinuclear inclusion body, and aggregates of intermediate filaments, including both vimentin and cytokeratins. MRTs have also been described in extrarenal organs including the large intestine [58], giving rise to rhabdoid colorectal Tumor (RCT) a rare, highly aggressive neoplasm frequently observed at the right colon of elderly patients [59, 60]. hSNF5/INI1 inactivation does not appear to be determinant in RCT development implying the involvement of other yet unknown loci. Its role in sporadic CRCs remains to be determined (Table 1). We have recently suggested that RCTs commonly present a combination of* BRAF *mutations, CIMP-high, and MSI-high phenotype [59, 60]. Further studies are needed to understand the complex interplay between chromatin remodelers CIMP tumors and CRC progression.

## 5. Epigenetic Changes and Epithelial **** Mesenchymal Transition in Colorectal ****Cancer Metastasis

Metastatic dissemination represents one of the key determinants of poor patients' prognosis in colon carcinogenesis. MSI-CRCs have been shown to be associated with a better prognosis than MSS-CRCs or CIN tumors [62], likely due to the lower metastatic potential, although the molecular bases have not been clearly established yet [62]. Among the processes involved in tumor invasion and metastasis, epithelial mesenchymal transition (EMT) has been proposed as a critical step in the acquisition of a more aggressive phenotype [62, 63]. EMT is a highly conserved process required for embryonic development, tissue remodeling, and wound repair. Activation of the EMT program in adult epithelia represses cell adhesion molecules (E-cadherin, cytokeratin, zona occludens 1 (ZO-1)) and induces mesenchymal markers (vimentin, N-cadherin, fibronectin) [64, 65]. Consequently, cells acquire a fibroblast-like appearance [64]. These biochemical and morphological changes enhance the migratory potential of cancer cells, promoting invasiveness, resistance to apoptosis, and synthesis of extracellular matrix components (ECM) [64–66]. Hepatocyte growth factor (HGF), epidermal growth factor (EGF), placental-derived growth factor (PDGF), or transforming growth factor-*β* (TGF-*β*) appears to be responsible for the induction or functional activation of a series of EMT-inducing transcription factors such as Snail, Slug, ZEB1, Twist, Goosecoid, and FoxC2 [64–69]. Their effects onto the EMT program depend upon the activation of a series of intracellular signaling networks involving ERK, MAPK, PI3K, AKT, SMADs, and integrins [70]. The WNT/*β*-catenin signaling pathway and E-cadherin loss are considered the major effectors of EMT and implicated in CRC metastatic progression [71–74]. The serrated pathway is associated with a lower frequency of nuclear *β*-catenin localization than the traditional one, suggesting that noncanonical Wnt/*β*-catenin pathways may influence metastasis formation especially in right-sided tumors (Figure 1(b)) [74, 75]. TGF-*β*1 has recently been demonstrated to induce EMT only in colon cancer cell lines bearing a wild type TGFBR2 receptor type II* (TGFBR2)* [76]. This gene contains an (A)10 repeat which is mutated in 80% of MSI-positive tumors, compared with just 0.6% of CIN tumors. Thus, it has been postulated that CIN-CRCs show an increased incidence of EMT and consequently shorter survival rates than MSI-tumors [76]. Recently, it has been suggested that the CIMP positive phenotype, in combination with MSI-CRCs, is usually associated with specific histological features and a poorer patients' prognosis, suggesting that the interplays of genomic instability with epigenetic variations are crucial events in metastatic dissemination [77, 78].

CRCs could then be classified according to their epithelial or mesenchymal subtype; by doing so, it would be possible to predict disease progression and recurrence even at early stages of tumorigenesis [79–81]. Consistent with this, cells that have undergone EMT histologically constitute cell buds that is single cells or small clusters of dedifferentiated cells at the invasive tumor front [82–85]. Tumor budding has been recognized as an independent prognostic factor to predict lymph node and distant metastasis or local recurrence and poor patient survival [82–85]. The EMT program has been identified as a possible unifying molecular signature in CRC clinical behavior and thus as a dominant pathway in metastatic progression (Figure 1(b)) [81]. According to this notion, our own data suggest that some CRC tumors presenting MSI, CIMP, and *BRAF* ^(V600E)^ mutation are extremely aggressive, characterized by a diffuse and intense EMT and resemble those originated via the serrated pathway (our unpublished data) [59, 60, 86]. These results support the existence of crosstalks between EMT and epigenetic modifications in the MSI-CRC group. That multiple pathways operate within apparently homogenous CRC subtypes cannot be excluded. 

## 6. Molecular and Phenotypic Heterogeneity of CRC 

Accumulating evidences indicate that human cancer cells harbor global epigenetic abnormalities and in CRC the number of genes inactivated by CpG island promoter hypermethylation is greater than the mutated genes [87, 88]. The three distinct pathways involving genomic instability (MSI, CIN, and CIMP) appear, in fact, to enhance the diversity of gene expression and phenotypic changes in CRC [87, 88]. They are generally present in a mutually exclusive fashion, rarely overlap, and provide a distinctive molecular signature of CRC development; for instance, MSI versus CIN or alternatively CIN versus CIMP (Figure 2(a)) [87, 88]. Sporadic cases with MSI can frequently be attributed to CIMP-related silencing of the mismatch repair gene MLH1 [89]. Some CRCs, however, present both MSI and CIN or no MSI or CIN at all: so it remains to be established whether they represent other subgroups [88, 90].

**Figure 3:**
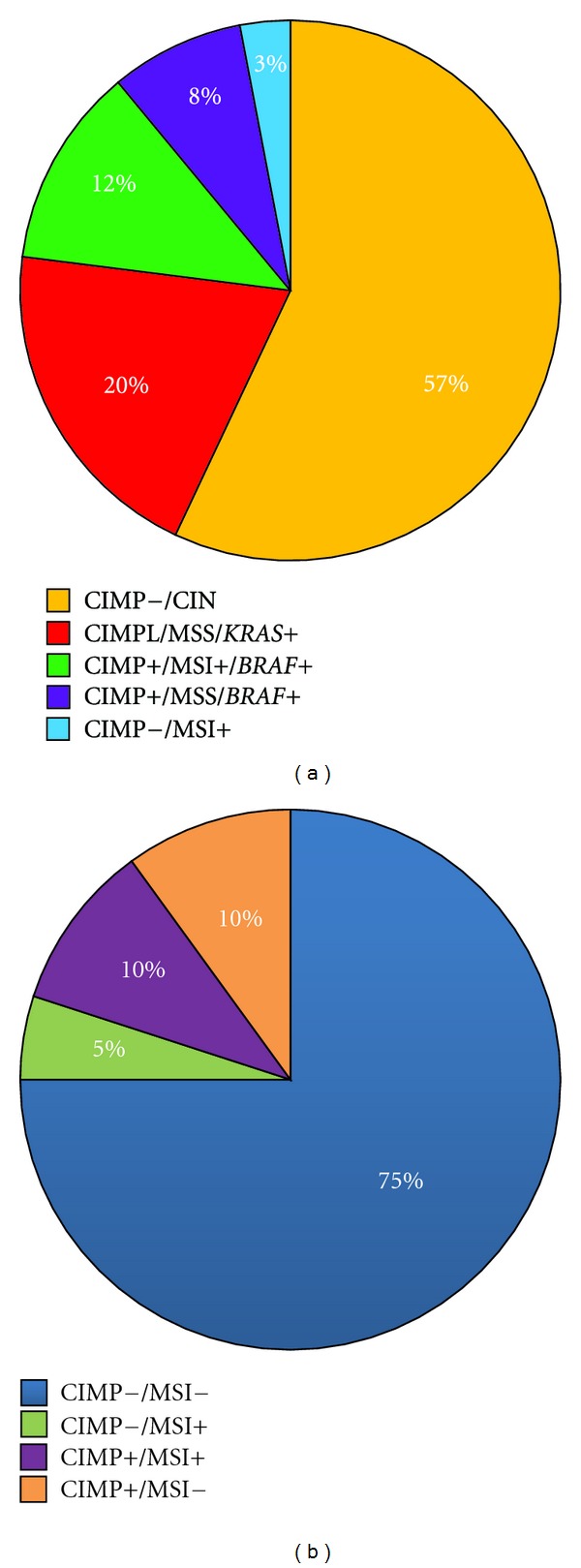
CRC classification in molecular subgroups. (a) Five molecular colorectal cancer subgroups have been proposed, stratified on the basis of genomic instability, presence/absence of CIMP [10]. The approximate frequencies of each CRC subtype in the Western population are illustrated. (b) Because the currently used criteria for CIN analysis are not uniform, as compared to MSI and CIMP, a classification into only 4 molecular subtypes has been proposed [77, 78]. The approximate frequencies in the Western population are illustrated. The distribution of the CIMP subgroup appears to correlate with ethnic differences (see text), suggesting that predisposition to the CIMP pathway may be tied to the contribution of yet unknown gene-environment interactions. MSI, microsatellite instability; CIMP, CpG Island methylator phenotype; CIN, chromosomal instability; CIMPL, CIMP Low.

Based on the stringent link between molecular, pathological, and clinical features, Jass classifies CRCs into 5 types [10], the approximate frequencies of each subgroup are reported in (Figure 3(a)). This classification is based on the correct identification of CIN, a survey that poses many problems because the currently used criteria are not uniform. It has been, then, proposed to classify CRCs into only 4 molecular subtypes taking into account MSI and CIMP only, as they are relatively more defined than CIN Figure 3(b) [77, 78]. Interestingly, the frequency of the CIMP+/MSI+ subtype versus CIMP−/MSI+ subtype differs substantially between Western and Asian populations [77]. People of Anglo-Celtic origin have a higher risk of cancers with CIMP and *BRAF *mutations than people of southern European origin [91, 92]. In line with this, in our CRC series of about 200 patients of southern European origin, *BRAF* mutations occur at a frequency even lower (≤5%) than that reported in the literature. Accumulating evidences suggest that CIMP+/MSI+ CRCs (10% of total) arise through the serrated pathway and are characterized by a high frequency of *BRAF *mutations (Figure 1(b)). We propose that, within the MSI-CRC group, the combination CIMP+/BRAF+ may constitute a new subtype associated with the worst clinical outcome [59, 60, 93]. Interestingly, the MSI+/CIMP+/BRAF+ subgroup is associated with synchronous colorectal cancers and exhibits a sarcomatoid dedifferentiation profile, due to the intense expression of mesenchymal markers [59, 60]. These data suggest that the variable predisposition observed among different populations to develop tumors through the serrated pathway may be tied to the specific contribution of yet unidentified gene-environment interactions. Further genetic and epigenetic studies of this pathway are needed to understand the reasons of these differences.

## 7. Final Remarks and Future Challenges 

The multistep model of colorectal tumorigenesis has been seminal and paradigmatic in cancer biology. One of the most intriguing but still unanswered questions is to understand the precise molecular events and their temporal occurrences that lead to tumor initiation, abnormal cellular expansion, and phenotypic changes. Likewise, the cell of origin and initial hit in a specific colon crypt compartment that determine the different CRC pathogenetic pathways remain obscure. Cancer stem cells have been proposed as the cellular drivers of subclonal expansion and probably vary in frequency and phenotypic features among different CRC pathways.

In this paper we have summarized the role of genetic/epigenetic changes in the origin of the multiple CRC pathways. Growing evidence suggests that the rate of epigenetic changes is estimated to be higher than that of genetic changes and could be a major determinant in the origin of tumor, clonal evolution, and tumor heterogeneity. These studies have, in fact, expanded our understanding of the pathogenetic mechanisms involved in tumor progression. The recognition and attempts to define the multiple pathogenetic pathways in CRC as reported here account for all these new accomplishments.

The identification and characterization of the CIMP positive phenotype, along with that of the full array of genes modified in CRC, have further deepened our knowledge and made even more complex the resulting picture. Whole epigenome sequencing supports the existence of a CIMP phenotype and epigenomic instability as a distinct molecular pathway prone to aberrant methylation in cancer. 

We hypothesize that the pathogenetic pathway of a specific CRC appears to be imposed at an early stage of the neoplastic evolution, through an “epigenetic memory” of the cell of origin. This condition may predispose the adult cancer cell to distinct degrees of epigenetic abnormalities, explaining the differences between the serrated and traditional pathway. So the acquisition of genomic and epigenomic instability (CIN, MSI, CIMP) are crucial features in tumor development. A better definition of the molecular mechanisms that initiate each of these alterations will be critical for the understanding of CRC pathogenesis and feasibility of targeting cancer cells specifically. In hereditary cancers, genomic instability can be attributed to mutations in DNA repair genes; however, the relationship between DNA repair systems, chromatin-remodelling complexes, and molecular basis of genomic instability in sporadic cancers remains unclear. Elucidation of these pathways will be relevant to improve the clinical management of patients. Age-dependent DNA methylation may drive gene expression changes associated with carcinogenesis. Elucidation of the link between age, environmental risk, and carcinogenesis will help to define the impact of epigenomic/genomic instability on multiple CRC pathways. These findings may have broad implications for cancer prevention, risk prediction, and prognosis. Moreover, they could provide new therapeutic targets or reliable biomarkers of chemo- or radiotherapy and ultimately could be a promising basis towards personalized therapeutic treatments.

## Figures and Tables

**Table 1:** Components of the SWI/SNF complex and their biological roles in colorectal cancer.

SWI/SNF components in mammals			
Members	Gene	Biological relevance	References
BRM	*SMARCA2*	(i) Mutated or lost in CRC cell lines	[45, 46, 49, 54]
		(ii) TSG	

BRG1	*SMARCA4*	(i) Mutated or lost in CRC cell lines	[48–50, 52, 53]
		(ii) EMT in CRC	
		(iii) TSG	

BAF170	*SMARCC2*	(i) CRC?	[46, 47]
		(ii) ESC self-renewal pluripotency	

BAF155	*SMARCC1*	(i) CRC?	[46, 47]
		(ii) ESC self-renewal pluripotency	

HLTF	*SMARCA3*	(i) TSG in CRC	[61]

BAF47	*SMARCB1*	(i) CRC?	[46, 55–58]
		(ii) TSG	

SWI: mating-type switching; SNF: sucrose nonfermenting; BRM: brahma; BRG1: brahma-related gene-1; BAFs: BRG- and BRM- associated factors; TSG: tumor suppressor gene; CRC: colorectal cancer; HLTF: helicase-like transcription factor; EMT: epithelial mesenchymal transition; ESC: embryonic stem cells. CRC? indicates a yet unknown role in colorectal cancer.

Note: representative SWI/SNF components are shown; at least 15 subunits have been described in mammals so far.
